# YBX1‐Mediated DNA Methylation‐Dependent *SHANK3* Expression in PBMCs and Developing Cortical Interneurons in Schizophrenia

**DOI:** 10.1002/advs.202300455

**Published:** 2023-05-21

**Authors:** Peiyan Ni, Chuqing Zhou, Sugai Liang, Youhui Jiang, Dongxin Liu, Zhicheng Shao, Haneul Noh, Liansheng Zhao, Yang Tian, Chengcheng Zhang, Jinxue Wei, Xiaojing Li, Hua Yu, Rongjun Ni, Xueli Yu, Xueyu Qi, Yamin Zhang, Xiaohong Ma, Wei Deng, Wanjun Guo, Qiang Wang, Pak C. Sham, Sangmi Chung, Tao Li

**Affiliations:** ^1^ The Mental Health Center and Psychiatric Laboratory State Key Laboratory of Biotherapy West China Hospital Sichuan University Chengdu 610041 P. R. China; ^2^ Department of Psychiatry McLean Hospital/Harvard Medical School Belmont MA 02478 USA; ^3^ Department of Cell Biology and Anatomy New York Medical College Valhalla NY 10595 USA; ^4^ Department of Neurobiology Affiliated Mental Health Center & Hangzhou Seventh People's Hospital Zhejiang University School of Medicine Hangzhou Zhejiang 310058 China; ^5^ NHC and CAMS Key Laboratory of Medical Neurobiology MOE Frontier Science Center for Brain Science and Brain‐Machine Integration School of Brain Science and Brain Medicine Zhejiang University Hangzhou Zhejiang 310058 China; ^6^ Department of Psychiatry Li Ka Shing Faculty of Medicine The University of Hong Kong Hong Kong, SAR 999077 China; ^7^ Centre for PanorOmic Sciences The University of Hong Kong Hong Kong, SAR 999077 China

**Keywords:** DNA methylation, cortical interneurons, induced pluripotent stem cells, schizophrenia, *SHANK3*, YBX1

## Abstract

Schizophrenia (SCZ) is a severe psychiatric and neurodevelopmental disorder. The pathological process of SCZ starts early during development, way before the first onset of psychotic symptoms. DNA methylation plays an important role in regulating gene expression and dysregulated DNA methylation is involved in the pathogenesis of various diseases. The methylated DNA immunoprecipitation‐chip (MeDIP‐chip) is performed to investigate genome‐wide DNA methylation dysregulation in peripheral blood mononuclear cells (PBMCs) of patients with first‐episode SCZ (FES). Results show that the *SHANK3* promoter is hypermethylated, and this hypermethylation (HyperM) is negatively correlated with the cortical surface area in the left inferior temporal cortex and positively correlated with the negative symptom subscores in FES. The transcription factor YBX1 is further found to bind to the HyperM region of *SHANK3* promoter in induced pluripotent stem cells (iPSCs)‐derived cortical interneurons (cINs) but not glutamatergic neurons. Furthermore, a direct and positive regulatory effect of YBX1 on the expression of *SHANK3* is confirmed in cINs using shRNAs. In summary, the dysregulated *SHANK3* expression in cINs suggests the potential role of DNA methylation in the neuropathological mechanism underlying SCZ. The results also suggest that HyperM of *SHANK3* in PBMCs can serve as a potential peripheral biomarker of SCZ.

## Graphical abstract

Induced pluripotent stem cell (iPSC) technology offers an approach to obtain functional brain tissues from patients with schizophrenia (SCZ). The consistent hypermethylation (HyperM) of *SHANK3* in peripheral blood mononuclear cells (PBMCs) and iPSC‐induced cortical interneurons (cINs) suggests the involvement of DNA methylation in the neuropathological mechanism of SCZ and its potential role as a peripheral biomarker for SCZ. 

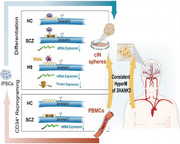

## 1 Introduction

Schizophrenia (SCZ), a debilitating neurodevelopmental disorder,^[^ ^1^ ^]^ is characterized by positive, negative, and cognitive symptoms and affects ~1% of the general population.^[^ ^2^ ^]^ Antipsychotic drugs have been the mainstream treatment for SCZ; however, their efficacy is considered satisfactory only for improving positive symptoms and limited for treating negative symptoms and cognitive impairment.^[^ ^3^ ^]^ Therefore, further understanding of the cellular and molecular mechanisms underlying SCZ pathogenesis is critical for developing novel mechanism‐based therapeutic strategies.

The heritability of SCZ is estimated to be up to 80%,^[^ ^4^ ^]^ implying that genetic factors play an important role in the pathogenesis of SCZ. The presence of common variants identified in genome‐wide association studies,^[^ ^5^ ^]^ including copy number variants^[^ ^6^ ^]^ and de novo mutations^[^ ^7^ ^]^ increases the genetic risk of developing SCZ. However, there are inconsistencies among studies on the incidence of SCZ in monozygotic twins,^[^ ^8^ ^]^ suggesting the involvement of non‐genetic risk factors in SCZ pathogenesis. Epigenetics, which can respond to environmental stimuli without altering the DNA sequence, could explain the risks associated with SCZ development that cannot be explained by genetic information alone.^[^ ^9^ ^]^

DNA methylation, an inherited epigenetic modification, has received increased attention in recent studies on SCZ pathophysiology.^[^ ^10^ ^]^ Methylome‐wide association studies (MWASs) of peripheral blood mononuclear cells (PBMCs) from patients with SCZ have identified several differentially methylated regions (DMRs).^[^ ^11^ ^]^ DNA methylation levels were shown to be associated with severity of the clinical symptoms^[^ ^12^ ^]^ and gray and white matter integrity deficits in patients with SCZ.^[^ ^13^ ^]^ Furthermore, pharmacotherapy can alter the DNA methylation status.^[^ ^14^ ^]^ These findings indicate that dysregulated DNA methylation in PBMCs of patients with SCZ may serve as a biomarker for SCZ.^[^ ^15^ ^]^

Postmortem samples have provided a detailed understanding of SCZ pathogenesis.^[^ ^16^ ^]^ Moreover, the development of single‐cell‐based techniques has allowed cell type‐specific analysis from postmortem brain samples.^[^ ^17^ ^]^ However, difficulties in obtaining early developmental tissues have hampered efforts to understand the developmental etiology of SCZ. Technological advances in induced pluripotent stem cells (iPSCs) have enabled us to access patient‐derived developmental cortical interneurons (cINs)^[^ ^18^ ^]^ and glutamatergic neurons (GNs),^[^ ^19^ ^]^ thus providing a model to analyze the developmental role of DNA methylation in different tissues.^[^ ^20^ ^]^ Furthermore, iPSC‐derived neurons/organoids allow us to explore the molecular mechanism regulating DNA methylation, including the identification of transcription factors (TFs) that directly bind to the gene promoter and regulate gene expression.^[^ ^21^ ^]^ Further understanding of the role of DNA methylation in SCZ may help in identifying specific biomarkers and aid in the diagnosis and treatment of this disease.

Herein, we analyzed the DNA methylation profile of PBMCs from patients with SCZ. Patients with first‐episode SCZ (FES) were included in our study to rule out the effect of possible confounders, such as antipsychotic drug usage, age at disease onset, and course of the disease. The cortical surface area and positive and negative syndrome scale (PANSS) scores were analyzed to explore their association with hypermethylation (HyperM) in the *SHANK3* promoter region and disease severity. To further explore the relationship between the identified HyperM regions in the *SHANK3* promoter in PBMCs and those in brain tissues, developmental cINs and GNs were generated from SCZ patient‐derived iPSCs and analyzed for cell‐type specific dysregulation of this identified region. Furthermore, potential TFs that bind to the candidate region with dysregulated DNA methylation were identified in developmental cINs using pull‐down and chromatin immunoprecipitation (ChIP) assays, followed by functional validation using shRNA‐mediated knockdown. HyperM in the *SHANK3* promoter was consistently observed in PBMCs and cINs, and a direct and positive regulatory effect of YBX1 on the expression of *SHANK3* was confirmed in developing cINs. The outcomes of our study could be exploited for developing peripheral biomarkers of SCZ.

## 2 Results

### 2.1 Genome‐Wide DNA Methylation in Peripheral Blood Mononuclear Cells (PBMCs) of Patients with FES

#### 2.1.1 Demographic and Clinical Characteristics

The demographic and clinical characteristics of all participants chosen for the methylated DNA immunoprecipitation‐chip (MeDIP‐chip) analysis are shown in **Table** **1**. In total, 22 patients with FES (11 males/11 females) and 20 healthy controls (HCs) (10 males/10 females) were recruited. No significant differences in age (*t* = 1.42, *p* = 0.16) or sex (*t* < 0.01, *p* = 1.00) were observed between the two groups. The results of the PANSS test are listed in Table 1.

**Table 1:** Demographic and clinical characteristics of subjects in the MeDIP‐chip

Characteristic	HC [*N* = 20]	FES [*N* = 22]	Analysis	
			*T*/*χ* ^2^ value	*p*‐value
Age (years), Mean ± SEM	23.35 ± 1.18	26.50 ± 1.82	1.42	0.16
Gender, n (%)	N/A	N/A	0.00	1.00
Male	10 (50.00)	11 (50.00)	N/A	N/A
Female	10 (50.00)	11 (50.00)	N/A	N/A
PANSS ts, Mean ± SEM	N/A	88.72 ± 3.68	N/A	N/A
PANSS ps, Mean ± SEM	N/A	25.50 ± 1.33	N/A	N/A
PANSS ns, Mean ± SEM	N/A	19.00 ± 2.36	N/A	N/A
PANSS gp, Mean ± SEM	N/A	44.22 ± 1.60	N/A	N/A

Note: HC: healthy control, FES: first‐episode schizophrenia, SEM, standard error of the mean; N/A: nonapplicable; PANSS: positive and negative syndrome scale; ts: total scale; ps: positive scale; ns: negative scale; gp: general psychopathology scale.

#### 2.1.2 Enrichment Peak Analysis

**Figure 1:**
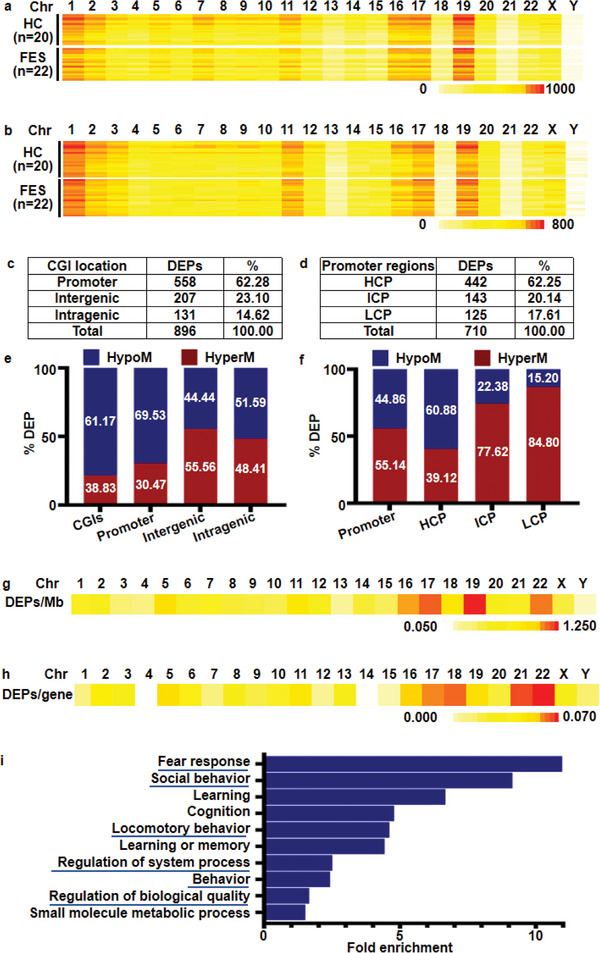
Genome‐wide DNA methylation profile of PBMCs from HCs and patients with FES. a,b) Heatmap showing EP distribution in CGIs (a) and promoter regions (b). The heatmap shows the number of EPs on each chromosome of all samples. c,d) DEP distribution in CGIs (c) and promoter regions (d). Table shows the number and proportion (%) of DEPs located in different CGIs or promoter regions. e,f) The proportion of HyperM and HypoM in CGIs (e) and promoter regions (f). g,h) Heatmap showing the average number of DEPs per Mb (g) or per gene (h). Heatmap depicting the number of DEPs in each chromosome. i) Top Ten GO terms enriched in genes annotated by DEPs in the promoters obtained using the GO database. The GO terms represent biological processes. Chr, chromosome; HC, healthy control; FES, first‐episode schizophrenia; CGI, CpG islands; DEP, differential enrichment peaks; HyperM, hypermethylation; HypoM, hypomethylation; GO, gene ontology.

The distribution of enrichment peaks (EPs) in the CpG islands (CGIs) of each chromosome is shown in the heatmap (**Figure** **1**a). Consistent with the MeDIP‐chip probe distribution (the number of probes targeting promoters accounted for 81.26%), the EPs were highly distributed in the promoter region of relevant transcripts, followed by the intergenic and intragenic regions. The distribution of EPs in the promoter region is shown in Figure 1b. The EP distribution pattern in CGIs and promoter regions of each chromosome was consistent between the FES and HC groups (Table S1, Supporting Information).

#### 2.1.3 Genome‐Wide Differential Enrichment Peak Analysis

Differential enrichment peaks (DEPs) represent DMRs in the FES and HC groups. As shown in Figure 1c, 896 DEPs were enriched in CGIs. Similar to the EP distribution, DEPs in CGIs were mainly concentrated in the promoter region (62.28%). Overall, 61.17% of the DEPs in CGIs were HyperM and 38.83% were hypomethylated (HypoM) (Figure 1e and Figure S1b, Supporting Information). Of 710 DEPs in the promoter regions, 62.25% were in high CpG density promoters (HCP) (Figure 1d). Furthermore, 44.86% of the DEPs located in the promoter region were HyperM, and 55.14% were HypoM in the FES group. Detailed results of the DEP analysis are summarized in Figure 1f and Figure S1c, Supporting Information. Considering that the length of chromosomes and the number of genes on each chromosome are different (Table S1, Supporting Information), the average number of DEPs in CGIs per Mb region (Figure 1g) and the number of DEPs in promoter per protein‐coding gene (Figure 1h) were calculated and displayed in the heatmap. Overall, both groups had a high‐density distribution on chromosome 22.

#### 2.1.4 Gene Ontology Analysis of Differential Enrichment Peaks (DEPs) Genes

The correlation between promoter HyperM and transcriptional repression is well established.^[^ ^22^ ^]^ Gene Ontology (GO) analysis of genes annotated by all DEPs in the promoters was conducted to understand the compromised biological processes in the FES group. The top ten GO terms of all DEP‐annotated genes (listed in Table S2, Supporting Information) and HyperM‐enriched GO terms (listed in Figure 1i) were highly correlated with the symptoms and possible pathogenesis of SCZ, such as fear response, social behavior, and locomotory behavior.

### 2.2 Hypermethylation (HyperM) of the *SHANK3* Promoter in the First‐Episode Schizophrenia (FES) Group

**Figure 2:**
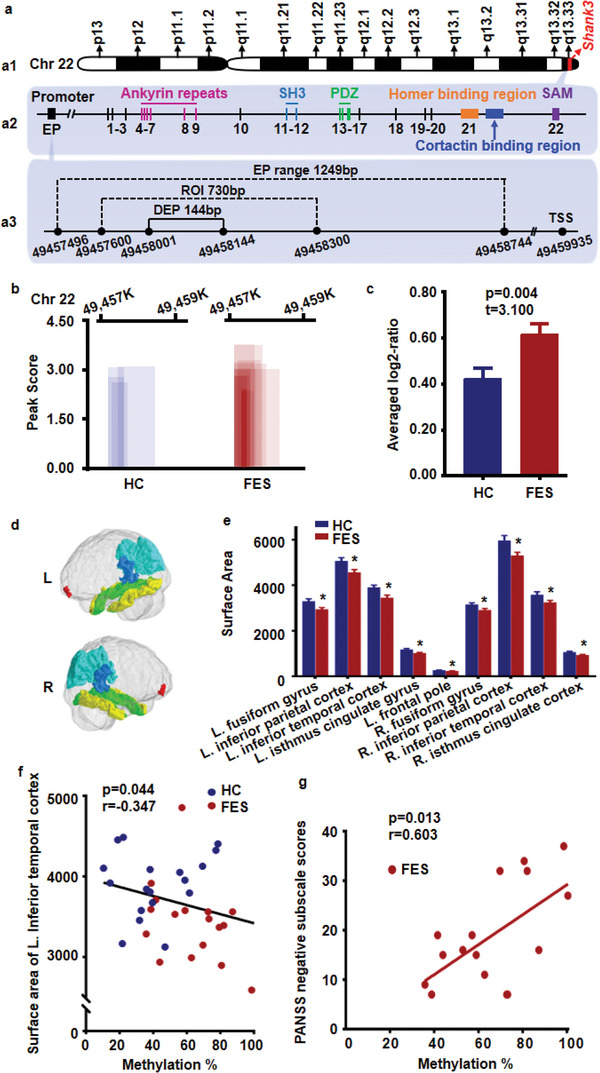
HyperM in the *SHANK3* promoter region in PBMCs of the FES group. a) Illustration showing the *SHANK3* gene location and protein structure along with experimental design for evaluating HyperM in the *SHANK3* promoter region. a1) Diagram of chromosome 22, p‐the short arm and q‐the long arm. a2) Structure of the *SHANK3* gene and the encoded protein. The number represents the exon. a3) The EP, DEP, and ROI in the *SHANK3* promoter. The number represents the base position on chromosome 22. b) EP length and scores in each subject (cut‐off value with peak score > 2.0). The horizontal coordinate denotes the location of the samples on chromosome 22. c) Averaged log2‐ratio with ROI analysis validated the HyperM in the FES group (*t* = 3.100, *p* = 0.004). Bar represents the mean ± SEM. d,e) The cortical surface area was reduced in the FES group. d) Green represents the fusiform. Light blue represents the inferior parietal cortex. Blue represents the isthmus cingulate. Red represents the frontal pole. Yellow represents the inferior temporal cortex. e) Significant regions with reduced surface area in the FES group compared to the HC group (*p* < 0.05). Student's *t‐*test was used for analysis and bars indicate the mean ± SEM. f) HyperM in the *SHANK3* promoter region was negatively correlated with the surface area of the left inferior temporal cortex (*p* = 0.044, *r* = −0.347). g) HyperM in the *SHANK3* promoter region was positively correlated with the PANSS negative subscale scores (*p* = 0.013, *r* = 0.603). EP, enrichment peaks; DEP, differential enrichment peaks; ROI, region of interest; L, left; R, right.

As listed in **Table** **2**, *SHANK3* presented the highest peak score among all genes annotated by DEPs in the promoter region, with a peak score cutoff of >2.0 and a peak differentially methylated (DM) value of <0.05. Furthermore, it is worth noting that *SHANK3* was present in six of the top ten GO terms of HyperM in the promoter region (underlined in Figure 1i). *SHANK3* is located on chromosome 22q13 (**Figure** **2**a1), which is considered a high‐risk region associated with SCZ pathogenesis.^[^ ^23^ ^]^ As shown in Figure 2a2, the SHANK3 protein contains SH3, PDZ, and SAM domains. SHANK3 interacts with a variety of synaptic proteins, including scaffold molecules, glutamatergic receptors, signaling proteins, and cytoskeletal proteins.^[^ ^24^ ^]^ The candidate EP region in the *SHANK3* promoter is 1249 bp long (Figure 2a3). The EPs in each sample over the cutoff value (peak score > 2.0) are shown in Figure 2b. The candidate DEP region was found to be 144 bp and HyperM in the FES group was validated by region of interest (ROI) analysis (Figure 2c, *t* = 3.100, *p* = 0.004).

**Table 2:** All genes annotated by DEPs in the promoter with peak DM value <0.05 and peak score >2.0

Gene name	Accession	Peak DM value	Peak score	Chr	Promoter classification	Strand	Peak start	Peak end	Peak length	Peak to TSS
HyperM in FES										
SHANK3	NM_001080420	0.036	2.61	chr22	HCP	+	49 458 001	49 458 144	143	−1862
ATP1A4	NM_001001734	0.049	2.55	chr1	LCP	+	158 412 293	158 412 454	159	−1636
TIMM13	NM_012458	0.047	2.40	chr19	HCP	−	2 380 432	2 380 591	249	283
REN	NM_000537	0.024	2.30	chr1	LCP	−	202 402 026	202 402 165	139	−7
SYNJ1	NM_003895	0.043	2.30	chr21	HCP	−	33 021 814	33 022 063	249	283
SYNJ1	NM_001160306	0.043	2.30	chr21	HCP	−	33 021 814	33 022 063	249	182
LTC4S	NM_145867	0.044	2.28	chr5	LCP	+	179 153 546	179 153 699	153	31
DAZL	NM_001351	0.028	2.23	chr3	HCP	−	16 621 961	16 622 124	163	−32
TRPV3	NM_145068	0.014	2.19	chr17	LCP	−	3 407 836	3 407 987	151	127
TMEM39B	NM_018056	0.027	2.18	chr1	HCP	+	32 311 113	32 311 260	147	97
MAPRE3	NM_012326	0.025	2.14	chr2	HCP	+	27 045 595	27 045 754	159	−1353
TDRG1	NR_024015	0.030	2.13	chr6	LCP	+	40 453 973	40 454 220	247	−43
SEMA3B	NM_004636	0.029	2.12	chr3	LCP	+	50 278 027	50 278 166	139	−1946
SEPT9	NM_001113496	0.029	2.06	chr17	LCP	+	72 958 552	72 958 811	259	474
HypoM in FES										
CPNE5	NM_020939	0.025	2.36	chr6	HCP	−	36 916 312	36 916 541	229	−1228
USP1	NM_003368	0.037	2.35	chr1	HCP	+	62 675 327	62 675 472	145	429
CNKSR2	NM_014927	0.040	2.35	chrX	HCP	+	21 302 586	21 302 745	159	−234
SLC2A4RG	NM_020062	0.019	2.33	chr20	HCP	+	61 841 862	61 842 103	241	328
C11orf35	NM_173573	0.014	2.20	chr11	HCP	−	551 581	551 746	165	−884
RASSF7	NM_003475	0.014	2.20	chr11	HCP	+	551 581	551 746	165	214
ADAM12	NM_003474	0.026	2.12	chr10	HCP	−	128 066 920	128 067 069	149	122
CDS1	NM_001263	0.016	2.11	chr4	HCP	+	85 723 364	85 723 491	127	347
STEAP2	NM_001040666	0.000	2.08	chr7	LCP	+	89 678 900	89 679 067	167	−2085
STEAP2	NM_001040665	0.000	2.08	chr7	HCP	+	89 678 900	89 679 067	167	−125
STEAP2	NM_152999	0.000	2.08	chr7	HCP	+	89 678 900	89 679 067	167	48
TRIM33	NM_033020	0.010	2.04	chr1	HCP	−	114 855 496	114 855 640	144	−264
ARHGAP21	NM_020824	0.010	2.01	chr10	HCP	−	25 052 392	25 052 527	135	143

Note: DM: different methylation; Chr: chromosome; TSS: transcription start site; HCP: high‐CpG‐density promoter; LCP: low‐CpG‐density promoter.

To evaluate the potential clinical significance of DNA methylation in the *SHANK3* promoter, its correlation with the cortical surface area and PANSS scores was analyzed. The cortical surface areas of different brain regions in the FES group exhibited a general decrease (HC, *n* = 18; FES, *n* = 18; Figure 2d,e and Table S3, Supporting Information). Furthermore, the area of the left inferior temporal cortex was negatively correlated with the methylation level of the ROI in the FES group (*r* = −0.347, *p* = 0.044, Figure 2f). Moreover, HyperM of the ROI was found to be specifically correlated with PANSS negative subscores (*r* = 0.603, *p* = 0.013, Figure 2g), but not total PANSS scores (*r* = 0.298, *p* = 0.261), positive subscores (*r* = 0.163 *p* = 0.548), or general subscores (*r* = 0.122, *p* = 0.647).

### 2.3 *SHANK3* Promoter HyperM in Human Induced Pluripotent Stem Cell (hiPSC)‐Derived Cortical Interneurons (cINs)

**Figure 3:**
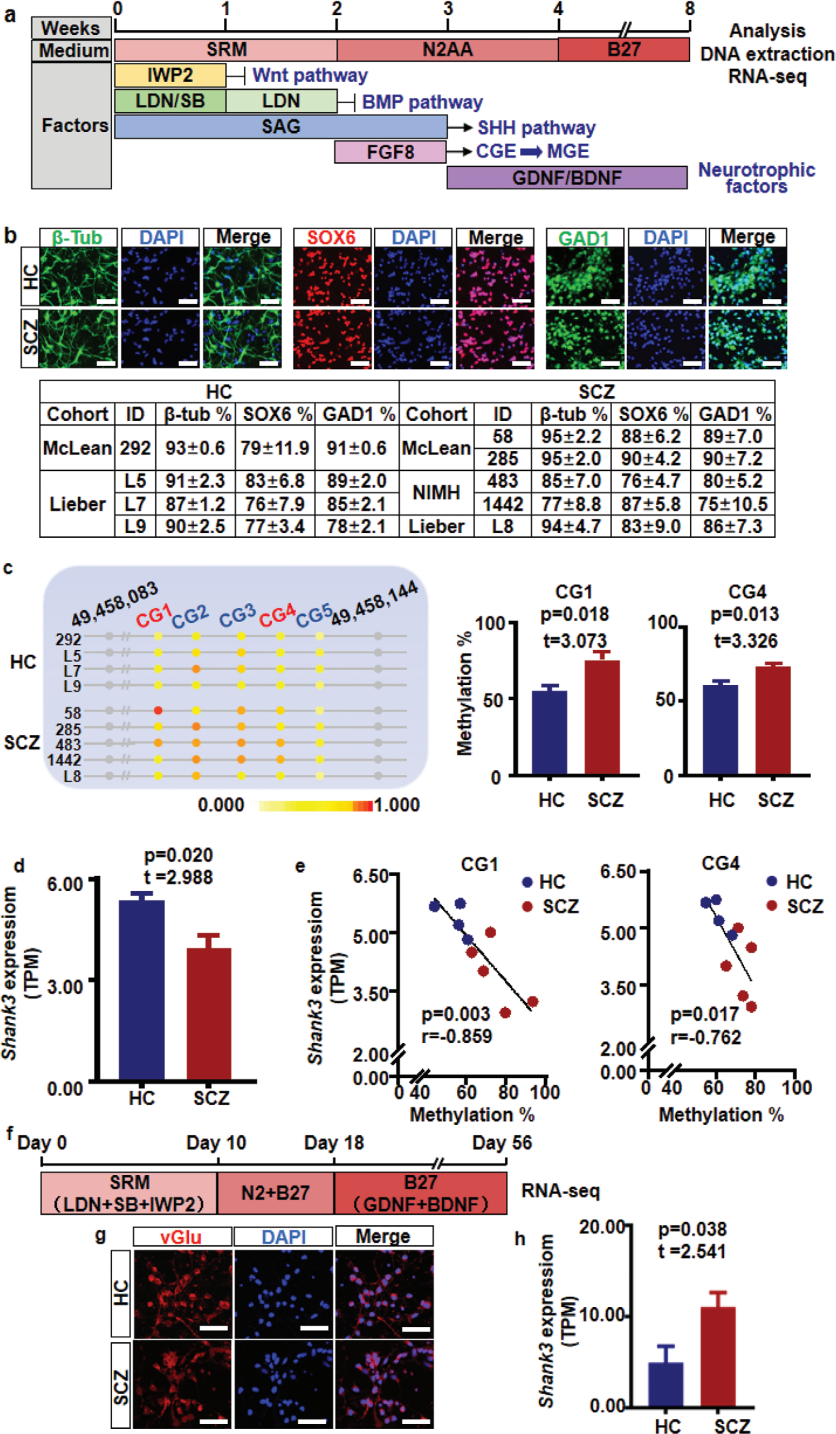
HyperM in the *SHANK3* promoter in iPSC‐derived cINs of the SCZ group. a) Differentiation scheme and experimental design to obtain cINs from iPSCs and ESCs. b) Immunocytochemistry and cell counting analysis of generated cINs for evaluating the expression of SOX6, GAD1, and *β*‐tubulin after 8 weeks of differentiation. Scale bar = 50 µm. Data are presented as the mean ± SEM from three independent experiments. c) CG1 and CG4 HyperM in the DEP of the *SHANK3* promoter was validated using the pyrosequencing method in cINs. Data are presented as the mean ± SEM (CG1, *t* = 3.073, *p* = 0.018; CG4, *t* = 3.326, *p* = 0.013). d) *SHANK3* expression in cINs was analyzed by RNA‐seq. Gene expression is shown as TPM. Data are presented as the mean ± SEM (*t* = 2.988, *p* = 0.020). e) The methylation of CG1 and CG4 was negatively correlated with the TPM of *SHANK3* (CG1: *r* = −0.859, *p* = 0.003; CG4: *r* = −0.762, *p* = 0.017). f) Differentiation scheme and experimental design for generating GNs from iPSCs. g) Immunocytochemistry analysis of generated GNs for evaluating the expression of vGlu and GABA at week 7 of differentiation. Scale bar = 50 µm. h) *SHANK3* expression in GNs was analyzed by RNA‐seq. Gene expression is shown as TPM. Data are presented as the mean ± SEM (*t* = 2.541, *p* = 0.038). SRM, serum replacement media; N2, N2 supplement (1:200); AA, 200 µm ascorbic acid; B27, B27 supplement (1:100); LDN, 100 nm LDN193189; SB, 10 µm SB431542; SAG, 0.1 µm smoothened agonist; IWP2, 5 µm inhibitor of Wnt production‐2; FGF8, 100 ng mL^−1^ fibroblast growth factor 8; BDNF, 10 ng mL^−1^ brain‐derived neurotrophic factor; GDNF, 10 ng mL^−1^ glial cell‐derived neurotrophic factor; HC, healthy control; SCZ, schizophrenia; *β*‐tub, *β*‐tubulin; TPM, transcripts per million.

As shown in Figure 2d–f, patients with FES exhibited abnormalities in the cerebral cortex area, which indicated that the neurodevelopmental abnormalities may play an important role in the pathogenesis of SCZ. Herein, the cINs and GNs, the main neuronal types of the cerebral cortex, were generated from subject‐derived iPSCs to explore the dysregulation of DNA methylation and expression of *SHANK3* in SCZ during development (HC, *n* = 6; SCZ, *n* = 7). Only male Caucasian subjects were recruited to reduce variations caused by ethnicity and sex, and patients treated with clozapine were recruited to include only those with a more severe and chronic disease^[^ ^18b^ ^]^ (**Table** **3**). There was no significant difference in the mean age of the two groups (*t* = 0.181, *p* = 0.861). As shown in **Figure** **3**a, developmental cINs were generated from patient‐derived iPSCs using a previously optimized protocol.^[^ ^25^ ^]^ This protocol efficiently produced homogeneous cINs, as validated by immunocytochemistry, where the majority of cells expressed the neuronal marker *β*‐tubulin and cIN markers SOX6 and GAD1 (Figure 3b). Differentiation efficiency was comparable between the two groups (SOX6, *t* = 1.938, *p* = 0.094; GAD1, *t* = 0.427, *p* = 0.681; *β*‐tubulin, *t* = 0.250, *p* = 0.810). The two CG loci were found to be HyperM in cINs derived from patients with SCZ (CG1, *t* = 3.073, *p* = 0.018; CG4, *t* = 3.326, *p* = 0.013, Figure 3c). RNA‐seq analysis showed *SHANK3* transcripts per million (Figure 3d). As shown in Figure 3e, the methylation of both CG1 and CG4 was negatively correlated with *SHANK3* expression (CG1, *r* = −0.859, *p* = 0.003; CG4, *r* = −0.762, *p* = 0.017). This negative correlation indicated that HyperM in the promoter region of *SHANK3* may affect the binding of certain TFs, which in turn perturbs *SHANK3* expression in SCZ. Unlike cINs, the expression of *SHANK3* was mildly upregulated in GNs of the SCZ group compared with GNs of the HC group (*t* = 2.541, *p* = 0.038, Figure 2f–h), suggesting cell type‐specific dysregulation of *SHANK3* in SCZ brain cells.

**Table 3:** Demographic and clinical characteristics of subjects in iPSC‐derived cINs and GNs

	Cohort	ID	Gender	Age	Race	Treatment	Differentiated neurons
HC	McLean	292	Male	43	Caucasian	None	cINs
		365	Male	52	Caucasian	None	GNs
	Lieber	L5	Male	54	Caucasian	None	cINs
		L7	Male	25	Caucasian	None	cINs, GNs
		L9	Male	38	Caucasian	None	cINs, GNs
	MGH	107	Male	27	Caucasian	None	GNs
SCZ	McLean	58	Male	43	Caucasian	Clozapine	cINs, GNs
		285	Male	47	Caucasian	Clozapine	cINs, GNs
	NIMH	483	Male	39	Caucasian	Clozapine	cINs
		1442	Male	26	Caucasian	Clozapine	cINs
		689	Male	32	Caucasian	Clozapine	GNs
		755	Male	32	Caucasian	Clozapine	GNs
	Lieber	L8	Male	50	Caucasian	Clozapine	cINs, GNs

### 2.4 YBX1 Directly Binds to the HyperM Region of the *SHANK3* Promoter and Positively Regulates Its Expression

**Figure 4:**
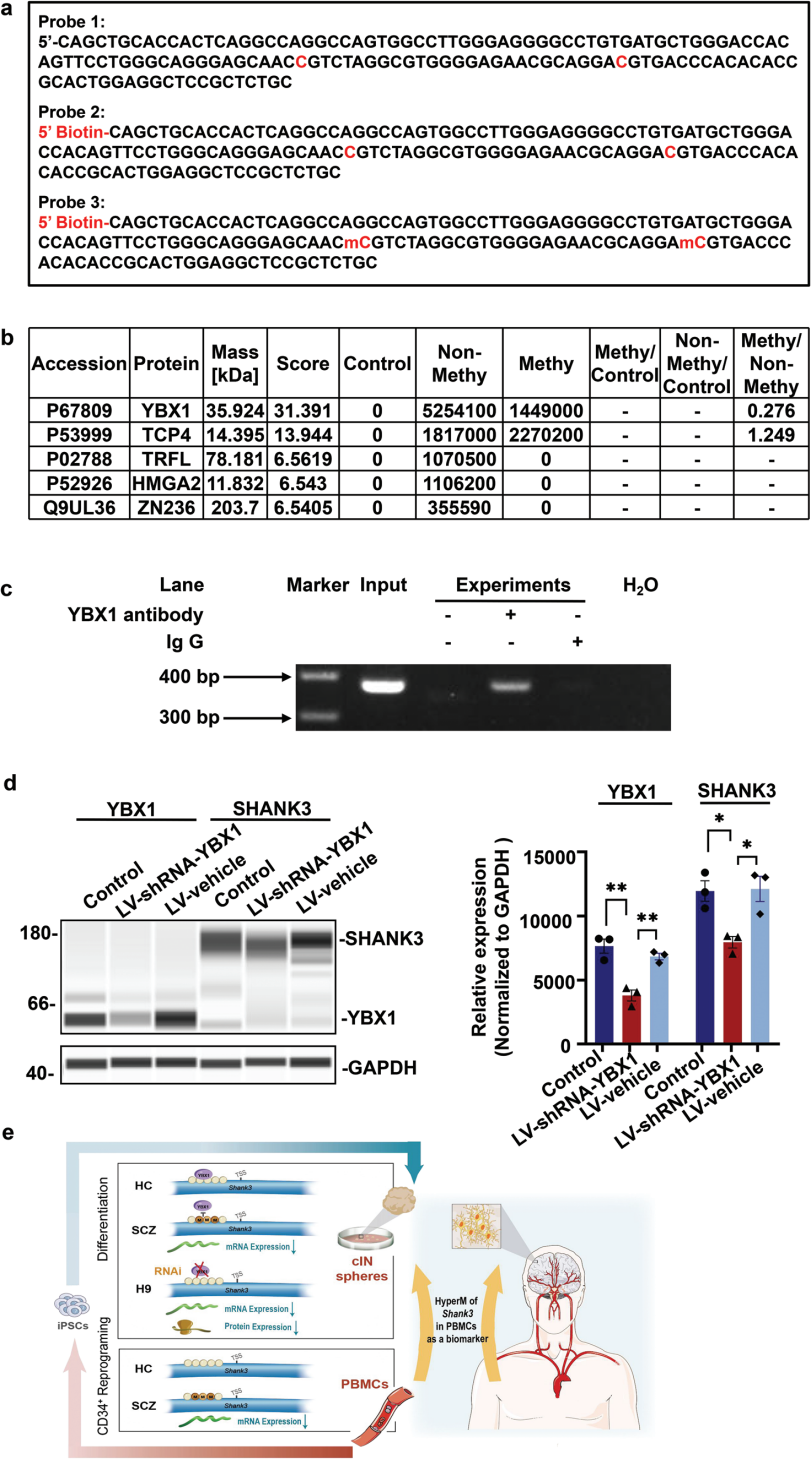
Binding of the transcription factor YBX1 to the HyperM region of the *SHANK3* promoter. a) Sequences of probes with biotin labeling and methylation modification used in the pull‐down experiment. Sequences without biotin‐labeling and methylation modification were used as negative controls. b) Transcription factors were identified by mass spectrometry analysis using the MaxQuant computational platform. c) ChIP analysis to confirm the direct binding of the transcription factor YBX1. Nonspecific IgG and beads‐only were used as negative controls. d) Capillary western blotting to evaluate YBX1 and SHANK3 expression in H9‐derived cIN spheres infected with vehicle or YBX1 shRNA lentiviruses. Data are presented as the mean ± SEM. One‐way ANOVA was used for analysis (*n* = 3) followed by Dunnett's post‐hoc analysis. After normalization to GAPDH, the relative expression of YBX1 (*F* = 22.434, *p* = 0.002; control vs shYBX1 Dunnett's *p* = 0.001; vehicle vs shYBX1 Dunnett's *p* = 0.004) and SHANK3 (*F* = 9.204, *p* = 0.015; control vs shYBX1 Dunnett's *p* = 0.016; vehicle vs shYBX1 Dunnett's *p* = 0.019) was quantified by measuring the peak areas detected by the Compass for Simple Western software. e) Schematic diagram illustrating the pathological role of the YBX1‐SHANK3 axis and the translational prospects of the *SHANK3* promotor HyperM in PBMCs in schizophrenia. HC, healthy control; SCZ, schizophrenia; cIN, cortical interneuron; PBMCs, peripheral blood mononuclear cells; HyperM, hypermethylation.

To identify the potential TFs, three probes (sequences listed in **Figure** **4**a) were designed to perform the pull‐down experiment: the control probe without label and modification and the biotin‐labeled probes with or without methylation modification in CG1 and CG4 loci. The probe‐bound proteins were analyzed using the MaxQuant computational platform and ranked based on the score (Figure 4b). YBX1 was on the top of the list (the typical peaks of YBX1 are shown in Figure S2b, Supporting Information). These results were further validated using the ChIP assay followed by PCR. The results confirmed the direct binding of the TF YBX1 to the HyperM region of the *SHANK3* promoter (Figure 4c). To investigate the regulatory role of YBX1 in cINs, shRNA‐mediated knockdown was performed. Capillary western blot analysis showed that YBX1 knockdown reduced *SHANK3* levels (Figure 4d), suggesting that YBX1 directly and positively regulates *SHANK3* expression. Altogether, these results suggest that reduced YBX1 binding to the HyperM *SHANK3* promoter region dysregulates *SHANK3* expression, which may contribute to SCZ pathogenesis.

## 3 Discussion

In this study, HyperM of the *SHANK3* promoter was observed in PBMCs from patients with FES but not in those from HCs (Figure 2b,c). Furthermore, the *SHANK3* promoter HyperM was negatively correlated with the cortical surface area of the left inferior temporal cortex (Figure 2f) and positively correlated with the PANSS negative subscores (Figure 2g). Corroborating previous studies,^[^ ^26^ ^]^ the FES group exhibited a significant reduction in the cortical surface area of multiple brain regions (Figure 2e). Grasby et al. provided evidence that genetic variation‐affected gene regulation in progenitor cell types during fetal development impacts adult cortical surface area, but not cortical thickness.^[^ ^27^ ^]^ Furthermore, Makowski et al. found a significant correlation between decreased temporal surface area and SCZ, which is consistent with our findings,^[^ ^28^ ^]^ suggesting that the developmental trajectories of the cortical surface in patients with SCZ are influenced primarily by early neurodevelopmental factors.^[^ ^29^ ^]^ Our results identified a specific correlation between HyperM in *SHANK3* and negative symptoms of SCZ, but not total PANSS scores (*r* = 0.298, *p* = 0.261), positive subscores (*r* = 0.163, *p* = 0.548), or general subscores (*r* = 0.122, *p* = 0.647). The negative symptoms of SCZ include interpersonal and social isolation that may impair the quality of life. Furthermore, the association between negative symptoms and neurodevelopment has also been reported in previous studies.^[^ ^30^ ^]^ Given the relevance of negative symptoms in SCZ prognosis and resistance to treatment, the development of novel and more efficient therapeutic strategies to treat these symptoms is urgently needed.^[^ ^31^ ^]^ However, the DNA methylation pattern in PBMCs and disease‐relevant neurons remains unexplored.

Patient‐derived iPSC‐derived cINs and GNs possess the same genetic makeup as the patient's brain cells and serve as a model to replicate the dysregulated neurodevelopmental process that can help in understanding the developmental mechanisms of SCZ. As shown in Figure 3d,h, decreased *SHANK3* expression was observed only in cINs, but not in GNs, and was negatively correlated with the *SHANK3* promoter HyperM. Studies on *SHANK3* expression in SCZ have been conducted using postmortem brain tissues; however, no significant differences were observed between the disease and healthy groups (BrainSeq: log FC = −0.038, *p* = 0.200, CommonMind: logFC = −0.010, *p* = 0.870).^[^ ^32^ ^]^ However, confounders that might affect gene expression in postmortem brain tissues should also be taken into account, such as the age at onset, course of the disease, postmortem sampling time, and pH of the samples.^[^ ^17^, ^32^ ^]^

SCZ diagnosis is mainly based on symptomatic information, which is severely constrained by subjectivity, symptom heterogeneity, and comorbidities.^[^ ^33^ ^]^ Since central nervous system abnormalities are reportedly mirrored in PBMCs,^[^ ^34^ ^]^ biological information from conveniently available PBMCs may be exploited as an alternative for symptom assessment and SCZ diagnosis. Encouragingly, our study indicates that HyperM of the *SHANK3* promoter in PBMCs can be a biological marker of decreased *SHANK3* expression in developmental cINs and could reflect the severity of negative symptoms and reduced cortical surface area (Figure 4e).

SH3 and multiple ankyrin repeat domains‐containing SHANK family proteins (also known as ProSAP) have been reportedly associated with autism spectrum disorder (ASD) and SCZ.^[^ ^7^, ^35^ ^]^ SHANK3 is a major postsynaptic scaffold protein that interacts with multiple proteins and complexes to orchestrate dendritic spines and modulate synaptic formation, maturation, and maintenance in excitatory neurons.^[^ ^35b^ ^]^ *SHANK3* deficiency in excitatory neurons disrupts the excitation/inhibition balance by affecting inhibitory synaptic transmission. Mechanistically, SHANK3 deficiency leads to a reduced number of synaptic puncta containing parvalbumin (PV) and compromised perineuronal nets containing Wisteria floribunda agglutinin,^[^ ^36^ ^]^ as well as reduced PV in the striatum.^[^ ^37^ ^]^ *SHANK3* is also expressed in PV‐^[^ ^38^ ^]^ and somatostatin (SST)‐expressing^[^ ^39^ ^]^ inhibitory interneurons. Moreover, the abnormal firing pattern in cultured cortical neurons of *SHANK3* knockout mice could be normalized by clonazepam, an enhancer of GABA‐mediated inhibitory transmission.^[^ ^40^ ^]^ Furthermore, excessive synaptic calcium signals and learning deficits in *SHANK3* mutant mice could be corrected by enhancing the expression of the NMDAR subunit GluN2B in SST interneurons.^[^ ^39^ ^]^ Thus, dysregulated *SHANK3* expression in cINs might play an important role in the pathogenesis of SCZ and may serve as a potential therapeutic target for SCZ treatment.

Although SHANK3 plays an important role in the pathogenesis of several neurodevelopmental disorders, studies investigating the regulation of *SHANK3* expression are limited.^[^ ^41^ ^]^ Our study identified that the TF YBX1 is a DNA methylation‐dependent regulator of *SHANK3* expression in developmental cINs derived from iPSCs. YBX1 is a multifunctional DNA/RNA‐binding TF that contains three domains: a cold shock protein (CSD) domain, an A/P domain, and a long C‐terminal domain.^[^ ^42^ ^]^ The CSD domain has been implicated in recognizing m5C‐modified mRNAs when YBX1 plays a role in epigenetic modification as an RNA 5‐methylcytosine reader.^[^ ^43^ ^]^ Studies have shown that YBX1 is involved in multiple biological processes,^[^ ^44^ ^]^ especially those related to nervous system development, neuronal differentiation, and synaptic transmission. Furthermore, YBX1 fine‐tunes the expression of polycomb repressive complex 2, a critical chromatin modifier that controls the execution of neurodevelopmental programs in neural progenitor cells,^[^ ^45^ ^]^ suggesting that YBX1 might influence neuronal differentiation and brain regionalization.

SHANK3 has been widely studied in ASD, and a strong association has been observed between *SHANK3* expression and core symptoms,^[^ ^46^ ^]^ causal mechanisms,^[^ ^47^ ^]^ and potential therapeutic prospects.^[^ ^41^, ^48^ ^]^ *SHANK3*, a risk gene for SCZ, has attracted wide attention,^[^ ^7^ ^]^ although its inherent mechanisms and therapeutic value have not been studied. In this study, we observed dysregulated DNA methylation in the *SHANK3* promoter in PBMCs from patients with SCZ and identified the regulatory mechanisms of methylation‐based epigenetic regulation of *SHANK3* expression in iPSC‐derived cINs. In this study, all the recruited patients for MeDIP‐chip and magnetic resonance imaging (MRI) were FES, which excluded the influence of the antipsychotic medications^[^ ^49^ ^]^ and the course of disease^[^ ^50^ ^]^ Patient‐derived iPSC‐induced developmental cINs used for exploring the pathogenesis of SCZ avoided some limitations of commonly used postmortem samples, such as the challenge of presenting the neurodevelopmental dysregulation^[^ ^51^ ^]^ and susceptibility to some confounders during sample preparation.^[^ ^52^ ^]^ The HyperM of *SHANK3* in PBMCs was consistent in patient‐derived iPSC‐induced neuronal tissues and correlated with clinical symptoms. Herein, the signature of HyperM of *SHANK3* in PBMCs may be utilized as a biomarker for SCZ pathogenesis. There are some limitations in this study to be mentioned. Although the statistical power was sufficient to make conclusions in the current studies,^[^ ^53^ ^]^ larger sample size and validation with independent replication samples would help increase the confidence. The cINs differentiated from patient‐derived iPSCs in our study mimicked neurons in the gestational stage, which is an important period for active cortical circuitry integration.^[^ ^54^ ^]^ Further mature neurons in vitro or neurons transplanted into the animal model in vivo are needed to understand the SHANK3‐related functional deficits in more mature cINs. Furthermore, future research should explore the *SHANK3*‐related mechanism of SCZ in stratified subgroups and further consider the specificity of cell type in postmortem with scRNA‐seq. In conclusion, the dysregulated expression of *SHANK3* in iPSC‐derived developing cINs implicates the involvement of DNA methylation in the neuropathological etiology of SCZ. Moreover, our findings proposed that the HyperM of *SHANK3* in PBMCs may provide a promising peripheral biomarker for SCZ.

## 4 Experimental Section

### Study Participants

Participants for whole‐genome DNA methylation screening (Table 1) were Han Chinese. This study was performed after obtaining the approval from the Institutional Review Board of West China Hospital, Sichuan University. Participants with a history of any major psychiatric disorder, substance abuse, neurological disorders, head trauma, or a family history of psychiatric disorders were excluded. The inclusion criteria were: (1) aged 18–60 years; (2) diagnosed with SCZ according to the trained psychiatrists using the Structured Clinical Interview for Diagnostic and Statistical Manual of Mental Disorders (fourth edition) Axis I Disorder, Patient Edition (SCID‐P) criteria;^[^ ^55^ ^]^ and (3) first‐episode of SCZ without any psychotropic medication treatment. The age at disease onset was recorded as the age at onset of psychotic symptoms as reported by the patients or their informants. All patients underwent the PANSS test^[^ ^56^ ^]^ and were followed up for at least six months to confirm the diagnosis. Finally, 22 patients with FES were recruited from the inpatient and outpatient psychiatric units of the West China Hospital. 20 HCs were screened using the non‐patient version of the SCID by trained psychiatrists. The study protocols involving iPSCs (HC, *n* = 6, SCZ, *n* = 7) were approved by the McLean Hospital/Partners Healthcare Institutional Review Board and New York Medical College Institutional Review Board. Experimental cohorts were chosen based on the authors’ selection criteria (Caucasian male patients treated with clozapine vs age‐ and gender‐matched Caucasian male HCs) without randomization to reduce variation caused by age, ethnicity, and gender. All recruited human fibroblasts were reprogrammed using the same modified RNA method^[^ ^57^ ^]^ by Cellular Reprogramming, Inc. (San Diego, CA). All procedures were performed according to the guidelines of the Institutional Review Board and all human samples were obtained with informed consent.

### Methylated DNA Immunoprecipitation‐Chip(MeDIP‐Chip): Microarray Hybridization

Genomic DNA from PBMCs was extracted and purified using the DNeasy Blood and Tissue Kit (Qiagen, Germantown, MD, USA). The MeDIP assay was performed as previously described.^[^ ^58^ ^]^ Briefly, DNA samples were sheared into 200–1000 bp fragments by sonication and immunoprecipitated using BioMag magnetic beads coupled to anti‐5‐methylcytidine mono‐antibodies for 12 h at 4 °C. Subsequently, the purified methylated DNA and the eluted input DNA were amplified and labeled with Cy5 and Cy3, respectively. The labeled DNA was then hybridized to the microarray slides. Human DNA Methylation 3× 720K Promoter Plus CpG Island Arrays (Roche NimbleGen, Madison, WI, USA) were used to identify methylated DNA regions, which were designed based on the HG38 genome release. Each array contained 27 728 CGIs annotated by UCSC and 22 532 well‐characterized RefSeq promoter regions (from ≈−2440 to +610 bp of the transcription start site) covered by ≈720 000 probes (probe distribution is shown in Figure S1a, Supporting Information). Scanning was performed using the Axon GenePix 4000B microarray scanner (Axon Instruments, Foster City, CA, USA).

### MeDIP‐Chip: Identification and Annotation of Methylated Regions

Raw data were extracted as paired files using NimbleScan software (Roche NimbleGen). The extracted data were further processed using the Bioconductor packages Ringo, Limma, and MEDME for median centering, quantile normalization, and linear smoothing. The enriched peaks were identified using a sliding‐window peak‐finding algorithm provided by NimbleScan v2.5 (Roche NimbleGen). Probes were selected as positive if their *p*‐value scores (after −log10 transformation using the Kolmogorov–Smirnov test with a 750 bp sliding window width) were above 2 (*p* < 0.01). A methylation peak was defined as a region with at least two consecutive positive probes (maximum spacing between nearby probes within the peak: 500 bp). The identified methylation peaks were mapped to genomic features of known transcripts defined in the UCSC Genome Browser HG38 RefSeq database. Mammalian genomes were punctuated by DNA sequences containing an atypically high frequency of CpG sites, termed CGIs. All CGIs could be grouped into three classes: promoter CGIs, intragenic CGIs, and intergenic CGIs. The promoters were subdivided into three classes: HCP, low CpG density promoters (LCP), and intermediate CpG density promoters (ICP).

### MeDIP‐Chip: DEP Processing

After normalization, the log2‐ratio values for each sample were averaged, and the *M*‐value for each probe was calculated to compare the differentially enriched regions of the two groups. The data were then scanned using the NimbleScan sliding‐window peak‐finding algorithm to find the DEP. The identified DEPs were mapped to the CGIs and promoters. Similar to the EP mapping, the mapped DEPs data in the CGIs were annotated as DEPs in the intergenic, intragenic, and promoter CGIs. The DEPs overlapping the promoter region of the transcripts were further annotated as DEPs in the HCP, ICP, and LCP. To support the reliability of DEPs in the *SHANK3* promoter, an ROI including eight probes within the EP region was analyzed. The mean log2‐ratio of individual probes covering defined regions was calculated to evaluate enrichment differences. For functional analysis, genes annotated by DEPs were searched in the GO database (http://www.geneontology.org), and the biological process domains in the GO results were analyzed. The *p*‐value denoted the significance of GO terms enrichment in the DEP genes.

### Magnetic Resonance Imaging (MRI) Analysis

Participants (HC, *n* = 18; FES, *n* = 18) who were selected for the MEDIP‐chip analysis underwent the MRI scan on a 3T MRI system (EXCITE; General Electric, Milwaukee, Wisconsin, USA) using an eight‐channel phased‐array head coil. Foam paddings and earplugs were used to minimize the head movement and scanner noise. A 3D spoiled gradient echo sequence (SPGR) was used to acquire high‐resolution T1 images from all subjects. The settings were as follows: TR = 8.5 ms, TE = 3.93 ms, flip angle = 12°, slice thickness = 1 mm, field of view = 24 × 24 cm^2^, matrix = 256 × 256, and voxel size = 0.47 × 0.47 × 1 mm^3^. A total of 156 axial images were obtained from each brain. Two experienced neuroradiologists reviewed the raw MRI data. No artifacts or gross anatomical abnormalities were observed in any subject.

Cortical reconstructions were performed using FreeSurfer (version 5.3.0; http://surfer.nmr.mgh.harvard.edu/fswiki/recon‐all/). This process included motion correction and averaging of the T1‐weighted images, removal of the non‐brain tissue, automated Talairach transformation, intensity normalization, tessellation of the gray matter/white matter boundary, automated topology correction, and surface deformation.^[^ ^59^ ^]^ This method used both intensity and continuity information from the entire 3D magnetic resonance volume in segmentation and deformation procedures to produce representations of the cortical surface area.

### Cell Culture

Human embryonic stem cell (hESC, H9 from WiCell Madison, WI, USA, passages 30–50) and hiPSC culture and cIN differentiation were performed as described previously. Patient‐specific hiPSCs were generated and characterized using previously published protocols.^[^ ^18^ ^]^ Thawed hESCs and hiPSCs were maintained on Matrigel (BD, San Jose, CA, USA)‐coated plates in Essential 8 (Invitrogen, Carlsbad, CA, USA) or ncEpic medium (Nuwacell Biotechnology, Hefei, Anhui, CN).

During embryonic development, cINs were produced from medial ganglionic eminence (MGE) progenitors by the action of relevant signaling molecules.^[^ ^25^, ^60^ ^]^ As shown in Figure 3a, MGE progenitors were generated in vitro as previously described.^[^ ^60^ ^]^ These MGE progenitors spontaneously differentiate to produce postmitotic cINs, a process similar to that observed during normal development. Once the MGE progenitors were established, all morphogenic signaling molecules were withdrawn from the culture. Glial cell‐derived neurotrophic factor (GDNF) and brain‐derived neurotrophic factor (BDNF) (both from Peprotech, Rocky Hill, CT, USA) were used to provide trophic support to postmitotic cINs. At 6 weeks of differentiation, cIN spheres were trypsinized in the presence of 0.1 m trehalose (Sigma, St. Louis, MO, USA) and passed through a cell strainer cap (35 µm nylon mesh, Corning, NY, USA) to remove dead cell clusters. Single cells were then plated on polyornithine (PLO; 15 mg mL^−1^; Sigma) and fibronectin (FN; 1 mg mL^−1^; Sigma)‐coated plates in B27GB media (DMEM‐F12 media with B27 supplement [1:100, Invitrogen] containing 10 ng mL^−1^ GDNF and 10 ng mL^−1^ BDNF) supplemented with 10 µm Y27632 (ApexBio, Boston, MA, USA) on the 1st day of culture only. After 3 days, the cells were fixed for immunocytochemistry. At 8 weeks of differentiation, the spheres were harvested in TRIzol (Invitrogen) for DNA and RNA isolation following the manufacturer's protocol. The DNA pellet was washed twice with 0.1 m sodium citrate (Sigma) and dissolved in 8 mm NaOH (Sigma). Both DNA and RNA were stored at −80 °C until further use.

GNs differentiation protocol was described in the authors’ previous publications.^[^ ^61^ ^]^ Briefly, iPSCs were trypsinized and cultured as floating spheres. From day 0 to day 10, the self‐assembled spheres were maintained in neuronal induction medium (DMEM‐F12 with 15% knockout serum replacement, 1% MEM‐NEAA, 100 µm *β*‐mercaptoethanol [all from Invitrogen], 100 nm LDN193189 [Stemgent, Cambridge, MS, USA], 10 µm SB431542 [Tocris Cookson, Ellisville, MO, USA], and 2 µm IWP2 [ApexBio]). Cells were then grown in neuronal differentiation medium (DMEM‐F12 with 0.25% N2, 0.5% MEM‐NEAA, 50 µm *β*‐mercaptoethanol [all from Invitrogen], and N21 MAX [R&D Systems, Minneapolis, MN]) from day 11. After 8 weeks, the spheres were trypsinized in the presence of 0.1 m trehalose (Sigma) and plated on PLO/FN‐coated plates in B27GB media for analysis.

### Methylation Validation by Pyrosequencing

Methylation of the CGIs in the DEP of the *SHANK3* promoter region in cINs was evaluated using pyrosequencing. In brief, 0.5 µg of genomic DNA from cINs was bisulfite‐treated using the DNA methylation kit (Zymo Research, Orange, CA, USA). Primers (blue‐highlighted sequences in Figure S2a, Supporting Information) were designed to target the CG loci surrounding the candidate DEP in the *SHANK3* promoter region. Bisulfite‐treated DNA was subjected to PCR amplification using the PyroMark PCR Kit (Qiagen) according to the manufacturer's protocol. The PCR amplification conditions were as follows: 95 °C for 10 min, followed by 45 cycles at 95 °C for 30 s, 56 °C for 30 s, and 72 °C for 30 s, and a final step of 72 °C for 10 min. After validation by agarose gel electrophoresis, the PCR product was subjected to quantitative pyrosequencing using PyroMark Q24 (Qiagen). Fully methylated and unmethylated human DNA samples (Zymo Research) were mixed to obtain standard curves. PyroMark Q24 software (Qiagen) was used to quantify the methylation level according to the following formula: methylation(%) = mC/(mC + C), where mC represented methylated cytosine levels and C represented unmethylated cytosine levels.

### RNA‐Seq Analysis

RNA quality was examined using 4200 TapeStation (Agilent Technologies, Santa Clara, CA, USA), and RNA concentration was determined using the Qubit Fluorometric Quantitation kit (Life Technologies, Carlsbad, CA, USA). For each sample, 100–200 ng of RNA was used to construct the cDNA sequencing library using the TruSeq Stranded mRNA Library Preparation Kit (Illumina, San Diego, CA, USA), following the protocol for polyadenylated RNA. Paired‐end sequencing (75 bp× 2) was performed using the NextSeq 550 system (Illumina). Raw sequence reads were demultiplexed and trimmed to remove adapters using Illumina bcl2fastq conversion software (v2.19, Illumina). Sequence reads of each sample were pseudoaligned to the human HG38 reference transcriptome, and transcript abundance was quantified using Kallisto.^[^ ^62^ ^]^ The RNA‐seq data are available on the GEO website (https://www.ncbi.nlm.nih.gov/geo/) under accession numbers GSE125805 for cINS and GSE184102 for GNs.

### Pull‐Down and Protein Validation by Mass Spectrometry

DNA fragments containing the candidate DEP of the *SHANK3* promoter region (with or without methylation modification in CG1 and CG4) were directly chemosynthesized in the pUC57 plasmid. Using these plasmids as templates, PCR was performed to amplify the chemosynthesized DNA fragments (yellow‐highlighted sequences in Figure S2a, Supporting Information). A biotin label was incorporated into the product during this process. After purification, the PCR products were used as probes in pull‐down experiments. Nuclear proteins were extracted from H9 cIN spheres using NE‐PER nuclear and cytoplasmic extraction reagents (Thermo Fisher Scientific, Waltham, MA, USA) according to the manufacturer's protocol. The biotin‐labeled or unlabeled probes were mixed with streptavidin magnetic beads (Thermo Fisher Scientific) and the extracted nuclear protein was added to the bead‐probe mixture. Nonspecifically bound proteins were collected using the lysis buffer (FitGene, Guangzhou, Guangdong, CN) and the specifically bound proteins were collected using the biotin‐streptavidin buffer (FitGene). The isolated proteins were analyzed by SDS‐PAGE and silver nitrate staining. Mass spectrometry was performed on the specifically bound proteins using the Q Exactive Orbitrap Mass Spectrometer (Thermo Fisher Scientific). Data analysis was performed using the MaxQuant computational platform (http://www.maxquant.org).

### Chromatin Immunoprecipitation (ChIP) Assay

ChIP analysis was performed using the ChIP Assay Kit (Millipore, MA, USA) according to the manufacturer's instructions. Briefly, cINs derived from H9 cells were cultured for 8 weeks, after which cINs were harvested and lysed using SDS lysis buffer (200 µL SDS lysis buffer/10^6^ cells). The lysates were sonicated (200 W for 5 s with 10‐s intervals, 100 cycles) on ice to produce DNA fragments of 200–1000 bp. The anti‐YBX1 antibody (Abcam, Cambridge, MA, USA) was used to immunoprecipitate the YBX1‐bound DNA. For the negative control, immunoprecipitation was performed either using IgG or without antibodies. The purified DNA samples were used as templates for PCR amplification. Primer sequences are shown in Figure S2a, Supporting Information.

### Lentivirus Construction and Cell Infection

Lentiviruses carrying the YBX1‐targeting shRNA were produced by subcloning the oligo sequence (5′‐GACGGCAATGAAGAAGATAAA‐3′) into the lentiviral expression vector GV 493 (hU6‐MCS‐CBh‐gcGFP‐IRES‐puromycin; GeneChem, Shanghai, Shanghai, CN). Negative control lentiviral particles with a scrambled sequence (5′‐TTCTCCGAACGTGTCACGT‐3′) were used as vehicles. Three to five H9 cIN spheres were infected with the indicated lentiviral particles (2E + 5 TU mL^−1^) for 48 h. For selection, the lentivirus‐infected spheres were cultured in the presence of 1 mg mL^−1^ puromycin for 96 h (Beyotime, Shanghai, CN). Then, the H9 cIN spheres were lysed in the RIPA buffer (Thermo Fisher Scientific) supplemented with a protease inhibitor cocktail (Roche, Basel, CH) on ice.

### Capillary Western Blot

Capillary western blot analyses were carried out using the chemiluminescent and fluorescent western blotting Jess system with a 12–230 kDa Jess separation module (ProteinSimple, Santa Clara, CA, USA) in accordance with the manufacturer's instructions. Protein concentration was measured using the BCA method (Vazyme, Nanjing, Jiangsu, CN). Protein samples were mixed with 0.1× sample buffer and 5× master mix (ProteinSimple) to achieve a final protein concentration of 1 µg µL^−1^ and then denatured at 95 °C for 5 min. Then, the denatured protein samples, blocking buffer, primary antibodies, HRP‐conjugated secondary antibodies, wash buffer, and chemiluminescent substrate (1:1 luminol‐peroxidase mixture) were added to specific wells of the assay plate. Rabbit anti‐YBX1 (1:10; Cell Signaling Technology, Boston, MA, USA), rabbit anti‐SHANK3 (1:10; Cell Signaling Technology), and rabbit anti‐GAPDH (1:50; HuaBio, Hangzhou, Zhejiang, CN) antibodies were used as primary antibodies. Anti‐rabbit antibodies (ProteinSimple) were used as secondary antibodies. After loading the plate, separation electrophoresis was performed in the capillary system and immunodetection was fully automated. The relative expression of proteins was automatically calculated using Compass for Simple Western software (version 5.0; ProteinSimple). All experiments were repeated three times.

### Statistical Analysis

All statistical analyses were performed using GraphPad Prism 8 (GraphPad Software, La Jolla, CA, USA) and SPSS version 22 (SPSS Inc., Chicago, IL, USA). Data normality and homogeneity of variance were tested using the Shapiro–Wilk test and Levine's test, respectively. A two‐tailed unpaired *t*‐test was used to compare the means of two groups when the assumptions of normal distribution and equal variance were met. When there were significant differences in the homogeneity of variance, a *t*‐test with Welch's correction was used. With age, sex, and years of education as covariates, a partial correlation was evaluated between PANSS scores and DNA methylation in patients with FES. A similar partial correlation analysis was performed between the cortical surface area and DNA methylation. When comparing more than two groups in the capillary western blot, one‐way analysis of variance (ANOVA) was used. Dunnett's test was used to compare each group with the control group as a post‐hoc analysis with adjustment for multiple comparisons. A *p*‐value < 0.05 was considered statistically significant.

All participants were chosen without randomization to reduce variations. The experimental cohort for MEDIP‐chip analysis and iPSCs generation was chosen based on the selection criteria as described above. The statistical power of the iPSC samples included in this study was >80% according to the recently reported method.^[^ ^53^ ^]^ For MRI analysis of surface areas, post‐hoc analysis by G*Power software showed an adequate statistical power >80%. Cell counting was performed in a blinded manner.

## Conflict of Interest

The authors declare no conflict of interest.

## Supporting information

Supporting Information

## Data Availability

The data that support the findings of this study are available from the corresponding author upon reasonable request.
