# Supplementary material for: YBX1‐Mediated DNA Methylation‐Dependent SHANK3 Expression in PBMCs and Developing Cortical Interneurons in Schizophrenia
Source: Adv Sci (Weinh). 2023 May 21;10(20):2300455. doi: 10.1002/advs.202300455 (PMC10369273; doi:10.1002/advs.202300455)
Supplement: Supplementary file 1 — Supporting Information [file ADVS-10-2300455-s001.pdf]

ADVANCED SCIENCE
Open Access

Supporting Information

for *Adv. Sci.*, DOI 10.1002/advs.202300455

YBX1-Mediated DNA Methylation-Dependent *SHANK3* Expression in PBMCs and Developing Cortical Interneurons in Schizophrenia

Peiyan Ni, Chuqing Zhou, Sugai Liang, Youhui Jiang, Dongxin Liu, Zhicheng Shao, Haneul Noh, Liansheng Zhao, Yang Tian, Chengcheng Zhang, Jinxue Wei, Xiaojing Li, Hua Yu, Rongjun Ni, Xueli Yu, Xueyu Qi, Yamin Zhang, Xiaohong Ma, Wei Deng, Wanjun Guo, Qiang Wang, Pak C. Sham, Sangmi Chung* and Tao Li*

ADVANCED SCIENCE
Open Access

Supporting Information

for *Adv. Sci.*, DOI 10.1002/advs.202300455

YBX1-Mediated DNA Methylation-Dependent *SHANK3* Expression in PBMCs and Developing Cortical Interneurons in Schizophrenia

Peiyan Ni, Chuqing Zhou, Sugai Liang, Youhui Jiang, Dongxin Liu, Zhicheng Shao, Haneul Noh, Liansheng Zhao, Yang Tian, Chengcheng Zhang, Jinxue Wei, Xiaojing Li, Hua Yu, Rongjun Ni, Xueli Yu, Xueyu Qi, Yamin Zhang, Xiaohong Ma, Wei Deng, Wanjun Guo, Qiang Wang, Pak C. Sham, Sangmi Chung* and Tao Li*

Figure S1. EP and DEP distribution in CGIs and promoter regions.

a. EP distribution in CGIs and promoter regions. CpG islands: atypically high frequency of CpG sites; Promoter: from approximately -10 kb to +0.5 kb around the transcription start site (TSS). Intragenic: From +0.5 kb around the TSS to the transcription end site. Intergenic: The remainder that does not fall into either promoter or intragenic. HCP, High-CpG-density promoter; ICP, Intermediate-CpG-density promoter; LCP, Low-CpG-density promoter. b-c. DEP distribution in CGIs (b) and promoter regions (c). % represents the proportion of HyperM and HypoM in different CGIs or promoter regions.

CGI, CpG islands; DEP, differential enrichment peaks; HyperM, hypermethylation; HypoM, hypomethylation.

Figure S1 *Related to Figure 1*

a

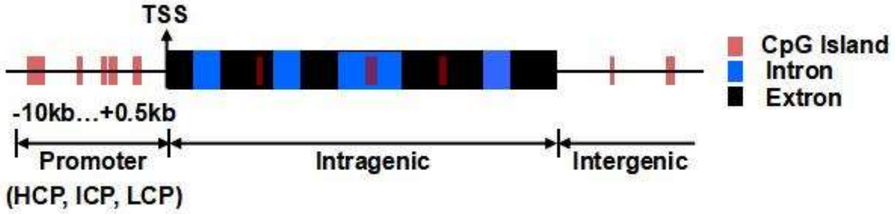

b

CGI location	HyperM		HypoM	
	DEPs	%	DEPs	%
Promoter	388	70.80	170	48.85
Intergenic	92	16.79	115	33.05
Intragenic	68	12.41	63	18.10
Total	548	100	348	100

c

Promoter regions	HyperM		HypoM	
	DEPs	%	DEPs	%
HCP	268	84.01	184	45.89
ICP	32	10.03	93	23.19
LCP	19	5.96	124	30.92
Total	319	100	401	100

Figure S2. Detailed DNA sequence for pyrosequencing, pull-down assay, and ChIP.

a. The DNA sequence and detailed information of the *SHANK3* promoter. Blue highlight: primers used for pyrosequencing; Yellow highlight: primers used for the pull-down assay; Green front: primers used for the ChIP-PCR; Red font: HyperM CG loci in the SCZ group. The number order of CG loci is limited to the interior of the pyrosequencing region. b. Proteins bound to the HyperM region of the *SHANK3* promoter were pulled down and analyzed by MS. The mass spectrum of YBX1 (m/z versus intensity) with identified list of relevant peaks.

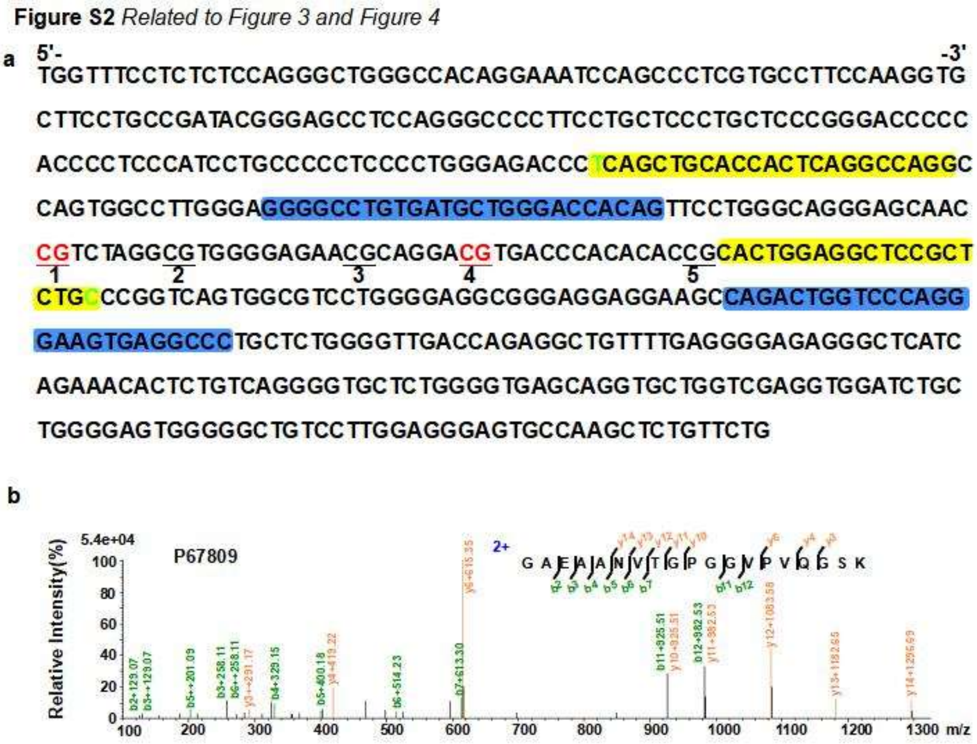

Table S1. The distribution of DEPs in the CGI region and promoter region on each chromosome. *Related to Figure 1*

Chr	Bases (GRCh38)	Protein-coding genes	DEPs in CGIs	DEPs in Promoter	Average DEPs in CGIs/Mb	Average DEPs in Promoter/protein-coding genes
1	248,956,422	2058	71	56	0.29	0.03
2	242,193,529	1309	63	48	0.26	0.04
3	198,295,559	1078	32	37	0.16	0.03
4	190,214,555	752	28	15	0.15	0.02
5	181,538,259	876	67	34	0.37	0.04
6	170,805,979	1048	37	38	0.22	0.04
7	159,345,973	989	48	27	0.30	0.03
8	145,138,636	677	31	23	0.21	0.03
9	138,394,717	786	28	24	0.20	0.03
10	133,797,422	733	25	26	0.19	0.04
11	135,086,622	1298	46	49	0.34	0.04
12	133,275,309	1034	34	27	0.26	0.03
13	114,364,328	327	15	11	0.13	0.03
14	107,043,718	830	23	17	0.21	0.02
15	101,991,189	613	18	15	0.18	0.02
16	90,338,345	873	55	35	0.61	0.04
17	83,257,441	1197	73	61	0.88	0.05
18	80,373,285	270	27	15	0.34	0.06
19	58,617,616	1472	71	57	1.21	0.04
20	64,444,167	544	15	17	0.23	0.03
21	46,709,983	234	13	14	0.28	0.06
22	50,818,468	488	39	34	0.77	0.07
X	156,040,895	842	31	28	0.20	0.03
Y	57,227,415	71	6	2	0.10	0.03

Chr: chromosome; DEP: differential enrichment peaks; CGI: CpG islands; Mb: megabase.

Table S2. Top 10 GO terms of DEP annotated genes. *Related to Figure 1g*

**All DEPs in promoter annotated genes**			
**GO ID**	**Term**	**Fold**	**p value**
GO:0042596	Fear response	10.96	4.17E-04
GO:0035176	Social behavior	9.13	1.91E-04
GO:0033555	Multicellular organismal response to stress	5.96	1.45E-03
GO:0051705	Behavioral interaction between organisms	5.71	1.76E-03
GO:0007626	Locomotory behavior	4.61	6.42E-05
GO:0007610	Behavior	2.43	2.45E-04
GO:0044057	Regulation of system process	2.50	3.41E-04
GO:0042592	Homeostatic process	1.76	9.56E-04
GO:0065008	Regulation of biological quality	1.65	1.00E-05
GO:0006950	Response to stress	1.42	7.84E-04
**HyperM DEPs in promoter annotated genes**			
**GO ID**	**Term**	**Fold**	**p value**
GO:0042596	Fear response	10.96	4.17E-04
GO:0035176	Social behavior	9.13	1.91E-04
GO:0007612	Learning	6.67	7.70E-06
GO:0050890	Cognition	4.78	3.42E-06
GO:0007626	Locomotory behavior	4.61	6.42E-05
GO:0007611	Learning or memory	4.43	3.96E-05
GO:0044057	Regulation of system process	2.50	3.41E-04
GO:0007610	Behavior	2.42	2.45E-04
GO:0065008	Regulation of biological quality	1.65	1.00E-05
GO:0044281	Small molecule metabolic process	1.51	1.64E-04
**HypoM DEPs in promoter annotated genes**			
**GO ID**	**Term**	**Fold**	**p value**
GO:0080090	Regulation of primary metabolic process	1.61	1.16E-09
GO:0031323	Regulation of cellular metabolic process	1.54	1.91E-08
GO:0019222	Regulation of metabolic process	1.49	5.56E-08
GO:0019219	Regulation of nucleobase-containing compound	1.71	6.04E-08
GO:0051171	Regulation of nitrogen compound metabolic process	1.54	1.05E-07
GO:0051252	Regulation of RNA metabolic process	1.66	9.11E-07
GO:0060255	Regulation of macromolecule metabolic process	1.47	9.81E-07
GO:0009889	Regulation of biosynthetic process	1.59	2.52E-06
GO:0031326	Regulation of cellular biosynthetic process	1.59	4.52E-06
GO:0032502	Developmental process	1.46	5.97E-06

Table S3. *Related to Figure 2e*

Cortical region	Surface area (Mean±SD)	T value	P value
Left fusiform gyrus	3122.361±422.723	-2.718	0.011
Left inferior parietal cortex	4818.583±612.536	-2.688	0.011
Left inferior temporal cortex	3680.444±498.653	-3.040	0.005
Left isthmus cingulate cortex	1100.417±169.104	-3.147	0.003
Left frontal pole	248.500±30.626	-3.272	0.002
Right fusiform gyrus	3032.667±313.461	-2.438	0.020
Right inferior parietal cortex	5638.167±854.137	-2.459	0.020
Right inferior temporal cortex	3415.361±500.684	-2.078	0.045
Right isthmus cingulate gyrus	998.667±139.430	-2.969	0.006
